# Induction of Autophagy Promotes Clearance of RHO^P23H^ Aggregates and Protects From Retinal Degeneration

**DOI:** 10.3389/fnagi.2022.878958

**Published:** 2022-06-30

**Authors:** Daniela Intartaglia, Giuliana Giamundo, Federica Naso, Edoardo Nusco, Simona Di Giulio, Francesco Giuseppe Salierno, Elena Polishchuk, Ivan Conte

**Affiliations:** ^1^Telethon Institute of Genetics and Medicine, Pozzuoli, Italy; ^2^Department of Biology, University of Naples Federico II, Naples, Italy

**Keywords:** Ezrin, RHO^P23H/+^, autophagy, retinal degeneration, ER-stress

## Abstract

Autophagy is a critical metabolic process that acts as a major self-digestion and recycling pathway contributing to maintain cellular homeostasis. An emerging field of research supports the therapeutic modulation of autophagy for treating human neurodegenerative disorders, in which toxic aggregates are accumulated in neurons. Our previous study identified Ezrin protein as an inhibitor of autophagy and lysosomal functions in the retina; thus, in turn, identifying it as a potential pharmacological target for increasing retinal cell clearance to treat inherited retinal dystrophies in which misfolded proteins have accumulated. This study aimed to verify the therapeutic inhibition of Ezrin to induce clearance of toxic aggregates in a mouse model for a dominant form of retinitis pigmentosa (i.e., RHO^P23H/+^). We found that daily inhibition of Ezrin significantly decreased the accumulation of misfolded RHO^P23H^ aggregates. Remarkably, induction of autophagy, by a drug-mediated pulsatile inhibition of Ezrin, promoted the lysosomal clearance of disease-linked RHO^P23H^ aggregates. This was accompanied with a reduction of endoplasmic reticulum (ER)-stress, robust decrease of photoreceptors' cell death, amelioration in both retinal morphology and function culminating in a better preservation of vision. Our study opens new perspectives for a pulsatile pharmacological induction of autophagy as a mutation-independent therapy paving the way toward a more effective therapeutic strategy to treat these devastating retinal disorders due to an accumulation of intracellular toxic aggregates.

## Introduction

Retinitis pigmentosa (RP) is a heterogeneous group of inherited retinal disorders (IRDs), affecting ~1.5 million people worldwide (Berson, 1996; Rattner et al., 1999; Hartong et al., 2006; Athanasiou et al., 2013). These conditions display genetic and clinical heterogeneity, with variable time of onset of the disease from childhood to middle age (Gu et al., 1999). Patients with this disorder typically develop night blindness and progressive loss of peripheral vision due to rod photoreceptor cell death. Secondary cone photoreceptor cell death causes legal blindness at later stages of RP. RP phenotypes can be generated by autosomal dominant, recessive or X-linked recessive modes, and ~60 genes have been implicated in RP disease (Farrar et al., 2002; Daiger et al., 2007). Although more than 60 RP genes have been identified to date, we are still far from an efficient therapy. At present, the recent success of clinical trials is providing sound evidence for the use of gene-based complementation therapeutic strategies to treat genetic recessive traits (Illing et al., 2002; Saliba et al., 2002; Mendes et al., 2005; Mendes and Cheetham, 2008; Mitter et al., 2012; Rodriguez-Muela et al., 2013; Mohlin et al., 2014). Among these studies, the successful reversal of blindness in individuals with Leber Congenital Amaurosis (LCA), a congenital form of IRD, represents a milestone result for treatment of genetic diseases and has been encouraging to the entire field of gene therapy (Mendes et al., 2005; Mitter et al., 2012; Chen et al., 2013; Rodriguez-Muela et al., 2013; Mohlin et al., 2014). Autosomal dominant retinitis pigmentosa (adRP) accounts for ~20% of all RP cases and is characterized by significant allelic and non-allelic heterogeneity. The difficulties posed by dominant genetic traits are owed to the nature of the disease. Typically, the dominant mutations affecting one allele cause complex sequels of events eventually leading to cell dysfunction and death through partial loss-of-function, gain-of-function, dominant negative, or toxic effects (Daiger et al., 2014). If one considers mutation-specific treatments, in the case of rhodopsin causing adRP, the theoretical number of therapeutics necessary to be designed and developed would be more than 200, compared to recessive genetic diseases in which one single correct copy of a gene can in principle cure different loss of function mutations. In addition, clinical studies for such numerous therapeutic protocols appear unrealistic and unfeasible. The recent advances in adRP have pointed out to the role of mistrafficking and accumulation of mutated and unfolded proteins in impairing normal cellular function and inducing toxicity in photoreceptor (PR) cells. Mutations in the *Rhodopsin* (*RHO*) gene, affecting the amino acidic sequence of the rod-specific protein rhodopsin, are responsible primarily for adRP and account for 30–40% of this form of RP. One of the most common causes of adRP is due to a missense mutation in the *RHO* gene, which results in the substitution of histidine for proline at amino acid residue 23 (RHO^P23H^) (Liu et al., 1996; Saliba et al., 2002). Mutated RHO^P23H^ results highly aggregation-prone within the ER leading to cellular stress and degradation by the ubiquitin–proteasome system (UPS) in response to the activation of unfolded protein response (UPR) (Illing et al., 2002; Saliba et al., 2002; Mendes et al., 2005). Importantly, autophagy is activated by ER-stress and contributes to the clearance of cellular debris in neurodegenerative disorders (Mendes and Cheetham, 2008; Mohlin et al., 2014). More importantly, autophagy has been shown to be an important housekeeping in the retina and specific deletion of the essential autophagy gene *Atg5* in rods induced retinal degeneration (Mitter et al., 2012; Chen et al., 2013; Rodriguez-Muela et al., 2013). The recent data implies autophagy as a complementary pathway for degradation of misfolded proteins in photoreceptor cells (Kunchithapautham and Rohrer, 2007). Moreover, current findings demonstrated an increase of autophagy flux in the inner segments of RHO^P23H/+^ rod photoreceptor cells, at onset and progression of retinal disease (Qiu et al., 2019), suggesting that it may be involved in the clearance of RHO^P23H^ protein in early stage of the disease or participating in the rod cell death at later stage. Interesting, valproic acid-mediated induction of autophagy in a RHO^P23H/+^ Xenopus laevis model demonstrated a protective effect of autophagy against retinal degeneration (Wen et al., 2019). On the other hand, increase of autophagy at PN14, corresponding to post symptom-onset stage, in which photoreceptors' death is already evident, worsen retinal degeneration in the RHO^P23H/+^ mice (Qiu et al., 2019), whereas inhibition of autophagy ameliorates retinal degeneration. These findings support the dual function of autophagy, suggesting a transition from a pro-survival to pro-apoptosis function in response to irreversible RHO^P23H^ accumulation and cellular stress. However, the protective role of the autophagy pathway in the photoreceptor cells is still largely unknown. In our previous research, we demonstrated that miR-211/Ezrin axis controls lysosomal biogenesis and function at the beginning of light–dark transitions in the retinal pigment epithelium (RPE)/PR cells and plays an important role in the activation of cell clearance. In line with these findings, pharmacological inhibition of Ezrin activity induced a daily pulse of autophagy in both RPE/PR cells rescuing imbalance of autophagy and retinal degeneration in miR-211^−/−^ mice (Naso et al., 2020). Importantly, this pharmacological therapy was safe and well-tolerated. Thus, the aim of this work was to assess the induction of selective autophagy *via* Ezrin inhibition as pharmacological therapy to rescue PRs' cell death from RHO^P23H^ accumulation and cellular stress at the onset of molecular and clinical symptoms of retinal degeneration; thus, in turn, blocking the transition of cell fate from survival to death. Our results show that a daily pulse of selective autophagy pathway in the RHO^P23H/+^ mice plays a protective role in promoting cell survival by reducing RHO^P23H^ accumulation and ER-stress, opening a new frontier for potential clinical application of autophagy activators as therapeutic compounds useful to counteract the onset and progression of retinal degeneration.

## Materials and Methods

### Animals and Ethics Approval Statement

The *P23H* mouse line (RHO^P23H/+)^ employed in this study was generated as previously described (Sakami et al., 2011). All studies on animals were conducted in strict accordance with the institutional guidelines for animal research and approved by the Italian Ministry of Health; Department of Public Health, Animal Health, Nutrition and Food Safety in accordance with the law on animal experimentation (article 31; D.L. 26/2014; protocol number: 0016304-21/07/2020-DGSAF-MDS-P). RHO^P23H/+^ mice were maintained on the C57Bl/6J background. In all experiments, we used as controls aged-matched littermates of RHO^P23H/+^ mice.

### Drug Treatments

Drug treatments were performed by once-daily intraperitoneal injection of *NSC668394*, at a dose of 0.226 mg/kg, as previously described (Celik et al., 2016).

### Electrophysiological Recordings (ERG)

Scotopic and photopic electrophysiological recordings were performed as described (Barbato et al., 2017). A National Instruments amplifier with a xenon Ganzfeld stimulator (CSO, Costruzione Strumenti Oftalmici, Florence, Italy) was used to record mice. Briefly, mice were dark-adapted for at least 3 h. Animals were anesthetized and positioned in a stereotaxic apparatus under dim red light. Their pupils were dilated with a drop of 0.5% tropicamide (Visufarma, Rome, Italy), and body temperature was maintained at 37.5°C. The electrophysiological signals were recorded through gold-plate electrodes inserted under the lower eyelids in contact with the cornea. The electrodes in each eye were referred to a needle electrode inserted subcutaneously at the level of the corresponding frontal region. The different electrodes were connected to a two-channel amplifier. For ERG analysis in dark-adapted conditions (scotopic), eyes were stimulated with light flashes. Five different light intensity stimuli were used ranging from 1 × 10^−4^ to 20 cd·s/m^2^. Amplitudes of *a*- and *b*-waves were plotted as a function of increasing light intensity. After the completion of responses obtained in scotopic conditions, the recording session continued with the purpose of dissecting the cone pathway through the photopic ERG. Photopic cone responses were isolated in light conditions with a constant background illumination of 50 cd·s/m^2^, with 10 flashes, and a light intensity of 20 cd·s/m^2^.

### Cell Culture and Treatments

HeLa cells were obtained from American Type Culture Collection (ATCC) and were cultured in Dulbecco's Modified Eagle Medium (DMEM) (Gibco) supplemented with 10% (v/v) FBS and 5% penicillin-streptomycin. Cell lines were maintained at 37°C, 5% CO_2_ in a humidified incubator according to the guidelines provided by the vendors. To analyze rhodopsin degradation, cells were plated for 24 h and then treated with 200-nM Bafilomycin A1 (Baf) (Sigma–Aldrich, B1793) for 3 h, 100-μg/ml cycloheximide (CHX) (Sigma–Aldrich, C4859), 100-mM Bortezomib (BTZ) (Sigma–Aldrich, 5043140001) and 10 μM of *NSC668394* or DMSO as previously reported (Bulut et al., 2012).

### Plasmids and Transfections

Cells were transfected at 70–80% confluence with 2.5 μg of pCS2-RHO P23H for 48 h. For plasmid transfection Lipofectamine 2000 (Invitrogen, 12566014) was used, following the manufacturer's protocol.

### Western Blot Analysis

Mouse eyes were enucleated and the RPE was separated from the retina. Cells were collected after transfections or treatments to extract total protein. Both mice and cell samples were lysed by using RIPA buffer (150-mM sodium chloride, 1% Triton X-100, 0.5% sodium deoxycholate, 0.1% sodium dodecyl sulfate, 50-mM Tris, pH 8.0) with inhibitors cocktail (Thermo Fischer Scientific, 78420). The concentration of total protein was determined by Bradford analysis and quantified by using a NanoDrop ND-8000 spectrophotometer (NanoDrop Technologies). Proteins were fractionated by sodium dodecyl sulfate–polyacrylamide gel electrophoresis (SDS–PAGE) and transferred to PVDF membranes (EMD Millipore, IPVH00010), then blocked in Tween 0.1%-Tris-buffered saline containing 5% non-fat milk or 5% bovine serum albumin (Sigma–Aldrich, 9048-46-8) for 1 h at room temperature and subsequently incubated overnight at 4°C with primary antibodies. For the Western blot analysis, the following antibodies were used: Mouse anti-Lamp1 (1:500, Sigma–Aldrich, L1418), mouse anti-Ezrin (1:1000, Novex, 357300), rabbit anti-phospho-Ezrin (Thr567) (1:700, Sigma-Aldrich, PA5-37763), rabbit anti-SQSTM1/p62 (1:1000, Sigma–Aldrich, P0067), rabbit anti-LC3 (1:1,000, Novus LC3B/MAP1LC3B), mouse anti-GAPDH (1:1000, Santa Cruz, SC-32233), rabbit anti-Perk (1:1,000, Cell Signaling, 3192), rabbit anti-BiP (1:1,000, Cell Signaling, 3177), rabbit anti-Xbp1s (1:1,000, Cell Signaling, 12782), mouse anti-Chop (1:1,000, Cell Signaling, 2895), mouse anti-RHO (1:500, Santa Cruz, sc-57432). After washing with 1% TBS, the membranes were incubated for 1 h at room temperature with the following secondary antibodies: Goat anti-rabbit IgG antibody, HPR conjugate and goat anti-mouse IgG antibody, HPR conjugate (1:10,000 EMD Millipore, 12–348; 12–349). Western blot detection was done with a GE detector (GE Healthcare Life Sciences) and quantified using ImageJ software.

### Immunofluorescence

Mouse eyes were fixed overnight in 4% paraformaldehyde in PBS at 4°C and then cryopreserved by treatment, first with 15% and then with 30% sucrose in phosphate-buffered saline and embedded in OCT. Twenty-micrometer cryosections were collected on slides (Superfrost Plus; Fisher Scientific, Pittsburgh, PA). Cells were fixed with 4% formaldehyde (Sigma–Aldrich) for 10 min at room temperature followed by washing with 1% PBS. After the fixation, the cells were permeated with blocking buffer (0.5% BSA, 0.005% saponin, 0.02% NaN_3_) for 1 h at room temperature. The following primary antibodies were used: rabbit anti-LAMP1 (1:400, abcam, ab24170), mouse anti-RHO (1:100, abcam, ab5417), rabbit anti-*c*-Arrestin (1:500, EMD Millipore, AB15282), rabbit anti-GFAP (1:400, Dako GA52461-2), rabbit anti-Iba-1 (1:1,000, Wako 019-19741). All incubations were performed overnight at 4°C. After washing with 1% PBS, slides were incubated with the following secondary antibodies: Alexa 594 goat anti-rabbit/mouse/rat (1:1,000, Invitrogen A-11037 rabbit, A-11032 mouse) or Alexa 488 goat anti-rabbit/mouse/rat (1:1,000, Invitrogen A-11008 rabbit, A-11001 mouse) and DAPI (1:500, Vector Laboratories H-1200) for 1 h at room temperature; then, the slides were washed with 1% PBS and mounted with PBS/glycerol and imaged with a Zeiss LSM700 microscope. All samples were imaged, and images were identically processed.

### Detection of Apoptotic Cell Death

The number of apoptotic cells was analyzed by TdT-mediated dUTP nick end labeling (TUNEL), using the *in situ* Cell Death Detection Kit, Fluorescein (Roche 11684795910) following the manufacturer's directions. Twelve-micrometer cryosections were collected on slides and subjected to TUNEL assay. To consider the presence of unspecific results, some retina sections were incubated with the reaction mix without TUNEL reaction enzyme. Sections were observed with a Leica DM-5500 microscope and then confocal images were acquired using the LSM700 Zeiss Confocal Microscopy system. The number of TUNEL-positive cells was evaluated in the dorsal and ventral part of the retina by manual counts with a Leica DM-6000 microscope, with the objective Leica ∞/0.17/D, HCX PL FLUOTAR, 40X/0.75 that has an area of 0.31 mm^2^.

### Image Analysis

#### Outer Nuclear Layer Thickness and OS Length Measurement

Measurements of ONL thickness and OS length were performed in the superior and inferior retina, located equidistant from the optic nerve head (ONH). Retina sections were analyzed by using a Leica DM-6000 microscope, with the objective Leica ∞/0.17/D, HCX PL FLUOTAR, 40X/0.75 that has an area of 0.31 mm^2^.

#### Cone Morphology Cell Counts

The number of normal cones/area was manually estimated in the superior and inferior retina, located equidistant from the ONH by using a Leica DM-6000 microscope, with the objective Leica ∞/0.17/D, HCX PL FLUOTAR, 40X/0.75 that has an area of 0.31 mm^2^. We matched the number of cone segments and dendrites clearly demarcated by the cone arrestin immunological staining.

#### GFAP and Iba-1 Quantification

Fluorescent images of dorsal and ventral region of mice retina were captured at 20X magnification using LSM700 Zeiss Confocal Microscopy system, converted to gray-scale and normalized to background staining, using ImageJ. Quantification of GFAP+ reactivity was measured as mean values to define fluorescence signal intensity (IntDen/Area) and as the area occupied by fluorescent-labeling in each region of interest.

To quantify the numbers of microglial cells, the number of Iba-1-positive cells was evaluated in the dorsal and ventral part of the retina by manual counts in each considered retinal layer: GCL, IPL, OPL, and ONL. These counts were pooled to obtain a mean number of microglial cells per layer, per retinal region and per animal group.

### Immunoelectron Microscopy Analysis

Hela cells were fixed with a mixture of 4% paraformaldehyde (PFA) and 0.05% glutaraldehyde (GA) for 10 min at room temperature, then washed with 4% PFA once to remove the residual GA and fixed again with 4% PFA for 30 min at room temperature. Next, the cells were incubated with a blocking/permeabilizing mixture (0.5% BSA, 0.1% saponin, 50-mM NH_4_Cl) for 30 min and subsequently with the primary monoclonal antibody anti-RHO, diluted 1:100 in blocking/permeabilizing solution. The following day, the cells were washed and incubated with the secondary antibody, the anti-mouse Fab fragment coupled to 1.4-nm gold particles (diluted 1:50 in blocking/permeabilizing solution) for 2 h at room temperature. The cells were then post-fixed as described in Polishchuk and Polishchuk (2019). After dehydration, the specimens were embedded in epoxy resin and polymerized at 60°C for 72 h. Thin 60-nm sections were cut on a Leica EM UC7 microtome. The EM images were acquired from thin sections using a FEI Tecnai-12 electron microscope equipped with a VELETTA CCD digital camera (FEI, Eindhoven, the Netherlands).

### Ribonucleic Acid Extraction, Retro-Transcription, and Quantitative Real-Time PCR

Total RNA was extracted from murine eyes using the miRNeasy Kit (QIAGEN 217004) according to the manufacturer's instructions. Ribonucleic acid was quantified using a NanoDrop ND-8000 spectrophotometer (NanoDrop Technologies). The cDNAs were generated using the QuantiTect Reverse Transcription Kit (Qiagen) for the qRT-PCR analysis. The qRT-PCR reactions were performed with nested primers (Table 1) and carried out with the Roche Light Cycler 480 system. The qRT-PCR reaction was performed using cDNA (200–500 ng), 10 μl of the SYBR Green Master Mix (ROCHE), and 400-nM primers, in a total volume of 20 μl. The PCR conditions for all the genes were as follows: Preheating, 95°C for 60 s; cycling, 45 cycles of 95°C for 10 s, 60°C for 10 s, and 72°C for 15 s. Quantified results were expressed in terms of cycle threshold (Ct). The Ct values were averaged for each triplicate. The HPRT gene was used as the endogenous control for the experiments. Differences between the mean Ct values of the tested genes and those of the reference gene were calculated as DCtgene = Ctgene – Ctreference. Relative expression was analyzed as 2-DCt. Relative fold changes in expression levels were determined as 2-DDCt.

**Table 1:** Primers used in qRT-PCR.

	**Sequence**
*Bcl2*	Forward primer CCTGACCCGGCTCCACT
	Reverse primer GATGGCAGCTCTTAGGACCC
*Fas*	Forward primer ATGAGATCGAGCACAACAGC
	Reverse primer TTAAAGCTTGACACGGCACCA
*Beclin*	Forward primer GGCCAATAAGATGGGTCTGA
	Reverse primer GCTGCACACAGTCCAGAAAA
*BiP*	Forward primer TTCAGCCAATTATCAGCAAACTCT
	Reverse primer TTTTCTGATGTATCCTCTTCACCAGT
*Chop*	Forward primer ATATCTCATCCCCAGGAAACG
	Reverse primer TCTTCCTTGCTCTTCCTCCTC
*Xbp1s*	Forward primer ACATCTTCCCATGGACTCTG
	Reverse primer TAGGTCCTTCTGGGTAGACC
*Atf4*	Forward primer GCCGGTTTAAGTTGTGTGCT
	Reverse primer CTGGATTCGAGGAATGTGCT
*Atf6*	Forward primer GTTACTCACCCATCCGAGTTGT
	Reverse primer CAACGTCGACTCCCAGTCTTC
*Lamp1*	Forward primer ATGTGTTAGTGGCACCCAGG
	Reverse primer TGTCTTCAGCTGGAAGTGGATGGT
*Lamp2*	Forward primer GTCTCAAGCGCCATCATACT
	Reverse primer TCCAAGGAGTCTGTCTTAAGTAGC
*Ctsd*	Forward primer AGGTGAAGGAGCTGCAGAAG Reverse primer ATTCCCATGAAGCCACTCAG
*Hprt*	Forward primer GTTGGGCTTACCTCACTGCT
	Reverse primer TCATCGCTAATCACGACGCT

### Statistical Analysis

All data are expressed as mean ± standard error unless indicated otherwise. The number of samples in each experiment is provided in appropriate methods description or figure legends. The statistical analysis was carried out at least from three independent experiments for each genotype and experimental conditions. In all experiments, the significance of differences between groups was evaluated and made using *t*-test. The *p*-values of significance are indicated where appropriate in the figure legends.

## Results

### *NSC668394* Administration Preserves Retinal Function in RHO^P23H/+^ Mice

**Figure 1:**
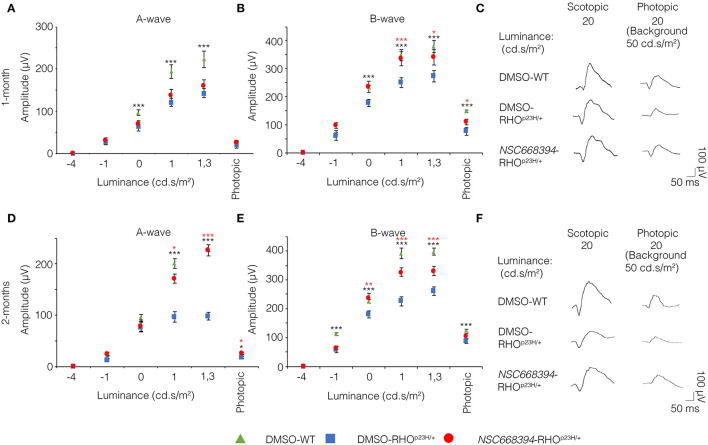
*NSC668394* RHO^P23H/+^ mice treatment results in progressive functional rescue of photoreceptors deficiency. **(A,B)** Representative ERG (*a*- and *b*-wave), plotted as a function of stimulus intensity, from 1-month old DMSO-treated WT (green triangle), DMSO-treated RHO^P23H/+^ (blue square), and *NSC668394*-treated RHO^P23H/+^ (red circle) mice. Error bars represent SEM. ***p ≤ 0.005, *p ≤ 0.05 *t*-test (DMSO-RHO^P23H/+^ *vs*. DMSO-WT; *NSC668394*-RHO^P23H/+^ *vs*. DMSO-RHO^P23H/+^). **(C)** Representative ERG (scotopic and photopic responses) traces of DMSO-treated WT, DMSO-treated RHO^P23H/+^, and *NSC668394*-treated RHO^P23H/+^ mice at 1-month of age. Scotopic indicates ashes of 20.0 cd·s/m^2^ light intensity; photopic indicates ashes of 20.0 cd·s/m^2^ light intensity on a constant background illumination of 50 cd·s/m^2^. **(D,E)** Representative ERG (*a*- and *b*-wave), plotted as a function of stimulus intensity, from 2 months-old DMSO-treated WT (green triangle), DMSO-treated RHO^P23H/+^ (blue square) and *NSC668394*-treated RHO^P23H/+^ (red circle) mice. Error bars represent SEM. ***p ≤ 0.005, **p ≤ 0.01, *p ≤ 0.05 *t*-test (DMSO-RHO^P23H/+^ *vs*. DMSO-WT; *NSC668394*-RHO^P23H/+^ *vs*. DMSO-RHO^P23H/+^). **(F)** Representative ERG (scotopic and photopic responses) traces of DMSO-treated WT, DMSO-treated RHO^P23H/+^ and *NSC668394*-treated RHO^P23H/+^ mice at 2 months of age. Scotopic indicates ashes of 20.0 cd·s/m^2^ light intensity; photopic indicates ashes of 20.0 cd·s/m^2^ light intensity on a constant background illumination of 50 cd·s/m^2^.

**Figure 2:**
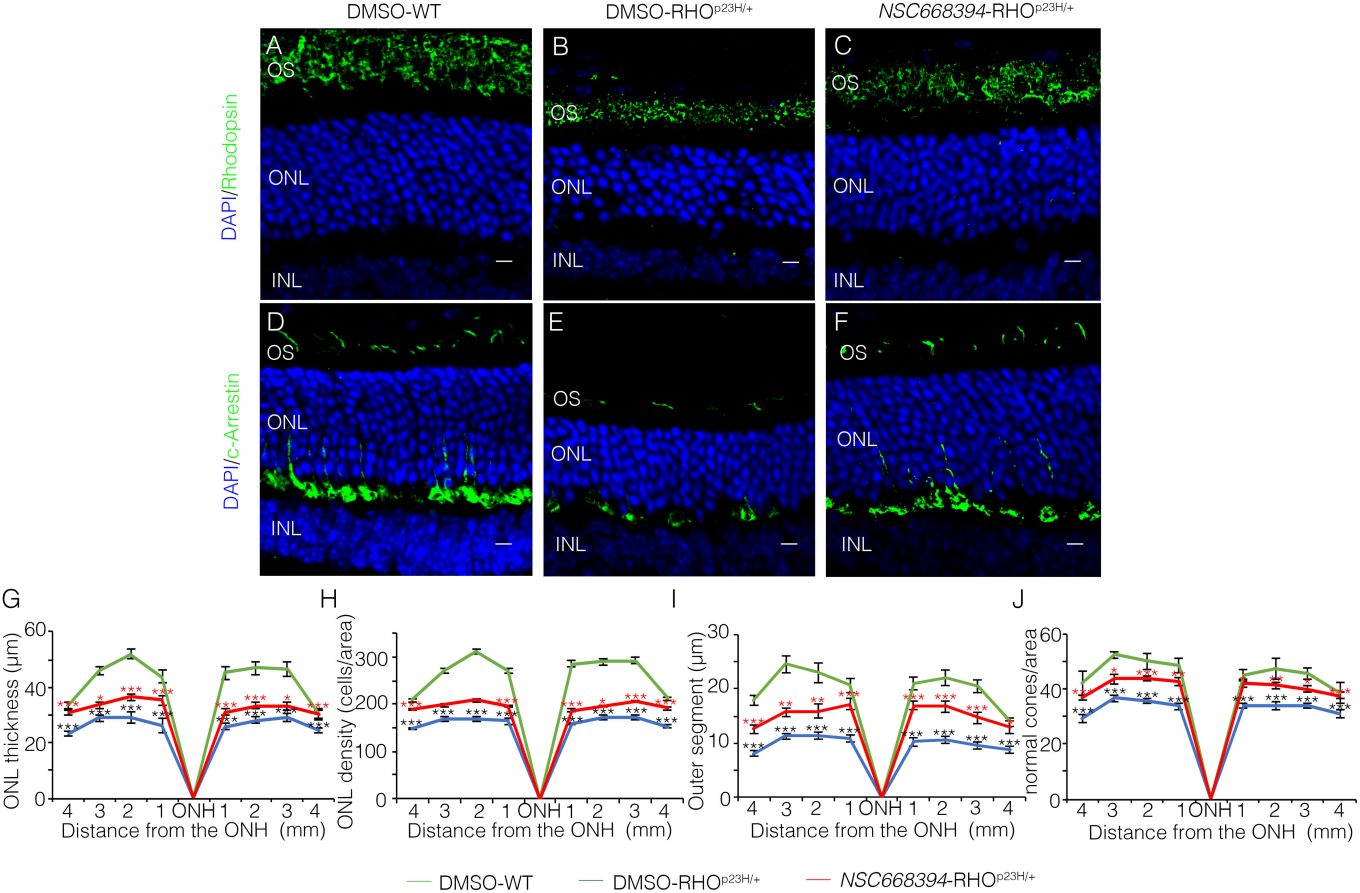
Two-months-old *NSC668394*-treated RHO^P23H/+^ mice show retinal morphology recovery. Representative images of retina cryosection immunostained with anti-Rhodopsin **(A–C)** and anti-*c*-Arrestin **(D,F)** antibodies from DMSO-treated WT **(A,D)**, DMSO-treated RHO^P23H/+^ **(B,E)** and *NSC668394*-treated RHO^P23H/+^ **(C,F)** mice at PN60. Nuclei are counterstained with DAPI (blue). At least *n* = 5 mice per group. Scale bar 10 μm. OS, outer segment; ONL, outer nuclear layer; INL, inner nuclear layer. **(G)** Graphs show the measure of ONL thickness from the retina of DMSO-treated WT (green line), DMSO-treated RHO^P23H/+^ (blue line), and *NSC668394*-treated RHO^P23H/+^ (red line) mice at 2 months of age. **(H)** Graphs show measure of ONL density (cells/area) from the retina of DMSO-treated WT (green line), DMSO-treated RHO^P23H/+^ (blue line), and *NSC668394*-treated RHO^P23H/+^ (red line) mice at 2 months of age. **(I)** Graphs show the reduction of OS length from 2 months of age onward from each analyzed retina region of DMSO-treated WT (green line), DMSO-treated RHO^P23H/+^ (blue line) and *NSC668394*-treated RHO^P23H/+^ (red line) mice. **(J)** Graphs show normal cone morphology (cones/area) from the retina of DMSO-treated WT (green line), DMSO-treated RHO^P23H/+^ (blue line), and *NSC668394*-treated RHO^P23H/+^ (red line) mice at 2 months of age. Error bars represent SEM. ***p ≤ 0.005, **p ≤ 0.01, *p ≤ 0.05 *t*-test. (DMSO-RHO^P23H/+^ *vs*. DMSO-WT; *NSC668394*-RHO^P23H/+^ *vs*. DMSO-RHO^P23H/+^).

**Figure 3:**
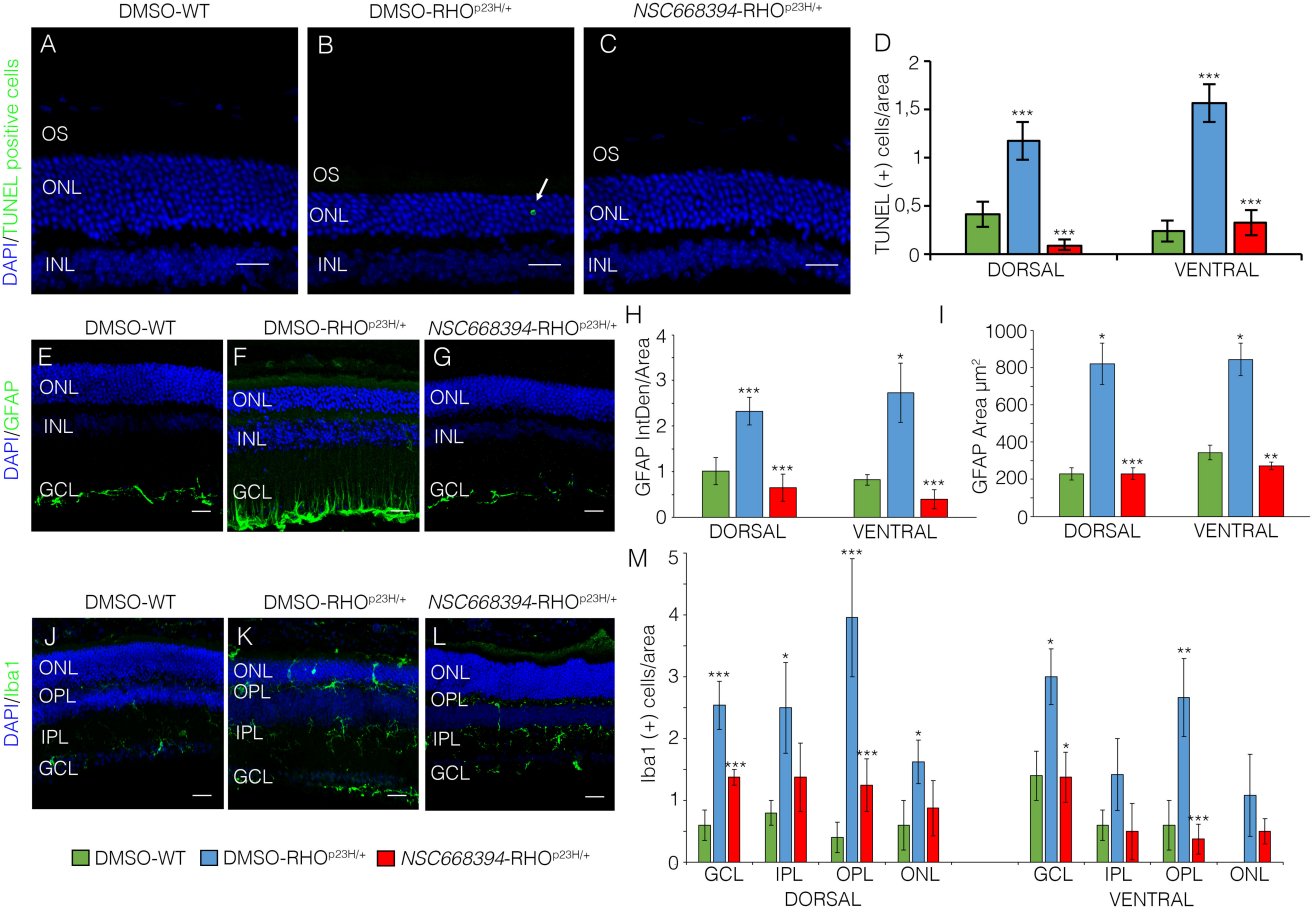
The *NSC668394*-treated RHO^P23H/+^ mice display a reduction in retinal cell death, microglia and inflammation. Representative images of DMSO-treated WT **(A)**, DMSO-treated RHO^P23H/+^ **(B)** and *NSC668394*-treated RHO^P23H/+^ **(C)** retinas of 2 months of age stained with TUNEL-fluorescein. Positive cells are indicated with white arrows. Nuclei are counterstained with DAPI (blue). At least *n* = 4 mice per group. Scale bar 20 μm. **(D)** Graph shows the number of TUNEL positive cells from the dorsal and ventral retina of DMSO-treated WT, DMSO-treated RHO^P23H/+^ and *NSC668394*-treated RHO^P23H/+^ mice at 2 months of age. Error bars represent SEM. Representative images of DMSO-treated WT **(E)**, DMSO-treated RHO^P23H/+^ **(F)** and *NSC668394*-treated RHO^P23H/+^ **(G)** retinas of 2 months of age stained with GFAP. Nuclei are counterstained with DAPI (blue). At least *n* = 3 mice per group. Scale bar 20 μm. **(H)** Bar graph is the quantitative analysis of optical density for GFAP fluorescence from the dorsal and ventral retina of DMSO-treated WT, DMSO-treated RHO^P23H/+^ and *NSC668394*-treated RHO^P23H/+^ mice at 2 months of age. Error bars represent SEM. **(I)** Bar graph shows quantification of GFAP-immunoreactive area in the dorsal and ventral retina of DMSO-treated WT, DMSO-treated RHO^P23H/+^, and *NSC668394*-treated RHO^P23H/+^ mice at 2 months of age. Error bars represent SEM. Representative images of DMSO-treated WT **(J)**, DMSO-treated RHO^P23H/+^ **(K)**, and *NSC668394*-treated RHO^P23H/+^ **(L)** retinas of 2 months of age stained with Iba-1. Nuclei are counterstained with DAPI (blue). At least *n* = 3 mice per group. Scale bar 20 μm. **(M)** Bar graph represents the number of Iba-1 positive (Iba-1+) cells in the dorsal and ventral retinal layers of DMSO-treated WT, DMSO-treated RHO^P23H/+^ and *NSC668394*-treated RHO^P23H/+^ mice at 2 months of age. Error bars represent SEM. OS, outer segment; ONL, outer nuclear layer; OPL, outer plexiform layer; INL, inner nuclear layer; IPL, inner plexiform layer; GCL, ganglion cell layer. ***p ≤ 0.005, **p ≤ 0.01, *p ≤ 0.05 *t*-test. (DMSO-RHO^P23H/+^ *vs*. DMSO-WT; *NSC668394*-RHO^P23H/+^ *vs*. DMSO-RHO^P23H/+^).

To assess whether pharmacological induction of autophagy exerts a protective effect when delivered in a fully differentiated retina before the molecular and clinical signs of PRs degeneration, we daily administered the Ezrin's inhibitor “*NSC668394*” in RHO^P23H/+^ mice from PN6 to PN60 using intraperitoneal injection of *NSC668394*, at a dose of 0.226 mg/kg as previously described (Celik et al., 2016). The starting point of intervention better represents the pre-symptomatic stage of the disease in human patients. Following the daily injection from PN6 onwards, we recorded an improved ERG responses at PN30 and PN60 in *NSC668394*-treated eyes *vs*. controls (Figure 1). Importantly, we recorded an amelioration in both scotopic *a*-wave and *b*-wave amplitudes at PN30 (Figures 1A,C), which persisted until PN60 (Figures 1D,F). Moreover, photopic ERG records, in the presence of background light, appeared higher amplitudes in *NSC668394*-treated animals compared to control (Figures 1C,F). Specifically, the *a*-wave amplitude of the RHO^P23H/+^ treated mice showed a trend of increase both at PN30 and PN60, albeit statistical significance was only observed at PN60 (Figures 1A,D). Notably, a significant increase in the *b*-wave amplitude was observed at both PN30 and PN60 (Figures 1B,E). Thus, we next sought to determine whether the pharmacological *NSC668394* treatment was indeed related to rescue of morphological and molecular detrimental cascade underlying PRs' ER-stress and cell death. Importantly, daily injections of *NSC668394* to RHO^P23H/+^ mice over two consecutive months (beginning at PN6) statistically ameliorated both outer nuclear layer (ONL) thickness and density in both dorsal and ventral portions of retina of the injected eyes, as detected by immunofluorescence assays (Figures 2A–H). First, we performed a morphometric analysis in PN18 retinas of *NSC668394*- and DMSO-treated RHO^P23H/+^ mice when the first evidence of alteration of the retinal structure appears (Supplementary Figures S1, S2). Importantly, *NSC668394*-treated RHO^P23H/+^ mice showed a relevant increase of the thickness of the ONL compared to DMSO-treated mice both in the dorsal and ventral retinal areas (Supplementary Figures S1A,G). Consistent with this finding, treatment of RHO^P23H/+^ mice with *NSC668394* resulted in a marked preservation of thickness of rod outer segments (OS), detected by immunostaining with anti-Rhodopsin antibody (Supplementary Figures S1A,C,H). Moreover, we measured a complete rescue of cone morphology in *NSC668394*-treated RHO^P23H/+^ that were like those recorded in DMSO-wild-type (WT) mice, as demonstrated by quantifying cones with cone segments (CS) and dendrites clearly labeled by immunostaining with anti-*c*-Arrestin antibody (Supplementary Figures S1D–F,I). Most importantly, we obtained similar beneficial effects also from *NSC668394*-treated RHO^P23H/+^ animals injected at PN6 and analyzed at PN60 (Figure 2), when retinal degeneration is advanced. Treatment of RHO^P23H/+^ mice with *NSC668394* led to an increased ONL thickness and density in both dorsal and ventral retina, as measured by DAPI staining and compared with DMSO-treated control mice (Figures 2A,H). Consistent with photoreceptor function assessments, significant recovery was also detected on the thickness of rod OS (Figures 2A–C,I), cones' morphology in *NSC668394*-treated RHO^P23H/+^ that were like those recorded in DMSO-WT mice (Figures 2D,F,J). Besides, we sought to determine whether the pharmacological *NSC668394* administration had an impact on PRs viability. Notably, treatment with *NSC668394* resulted in a significant decreased number of TUNEL-positive photoreceptor cells compared to DMSO-treated mice, supporting a reduction in PRs' cell death (Figure 3, Supplementary Figure S2). This outcome was significant in Ezrin inhibited- RHO^P23H/+^ mice at PN18 (Supplementary Figure S2), but it became even more consistent in treated retina at PN60 (Figures 3A,D). Next, we investigated possible inflammatory effects of Ezrin inhibition in the RHO^P23H/+^ retinae. For this purpose, we performed immunofluorescence staining for glial fibrillary acidic protein (GFAP) and ionized calcium-binding adapter molecule 1 (Iba-1) of RHO^P23H/+^ mice at PN60 (Figures 3E,M). Firstly, to measure activation of retinal gliosis, we quantified the fluorescent integrated density (IntDen) and the area of GFAP immunoreactivity in dorsal and ventral region of the retina. In RHO^P23H/+^ mice GFAP immunoreactivity was significantly higher than in the age-matched WT control and it extended to the ONL, indicating Müller cells activation and retinal gliosis. Remarkably, *NSC668394* treatment rescued gliosis as demonstrated by reduction of area of the GFAP immunoreactivity and IntDen (Figures 3E,I). In support of beneficial effect of the NSC668394 administration in RHO^P23H/+^ mice, we also observed a reduction of the number of microglia cells in the RHO^P23H/+^ retinal cryosections. We quantified the number of microglia cells by Iba-1 immunostaining, hallmark of activated microglial cells and retinal inflammatory response. Under DMSO treatment, RHO^P23H/+^ mice showed over twice as many Iba-1-positive cells compared to WT retinal cryosections, confirming that the RHO^P23H^ accumulation in rods induced cell death and secondarily strong activation of retinal microglia, which was localized in the ONL, inner nuclear layer (INL) and ganglion cell layer (GCL). Consistent with protective effects, *NSC668394* decreased Iba1-positive cell number in *NSC668394*-RHO^P23H/+^ treated animals compared to DMSO controls (Figures 3J,M). This result supports that the protective effect of *NSC668394* in RHO^P23H/+^ mice may be mediated by the clearance of unfolded RHO^P23H^ aggregates *via* targeting Ezrin activity and induction of autophagy pathways.

### *NSC668394*-Mediated Inhibition of Ezrin Reduces Protein Accumulation *via* Lysosomal Activity

**Figure 4:**
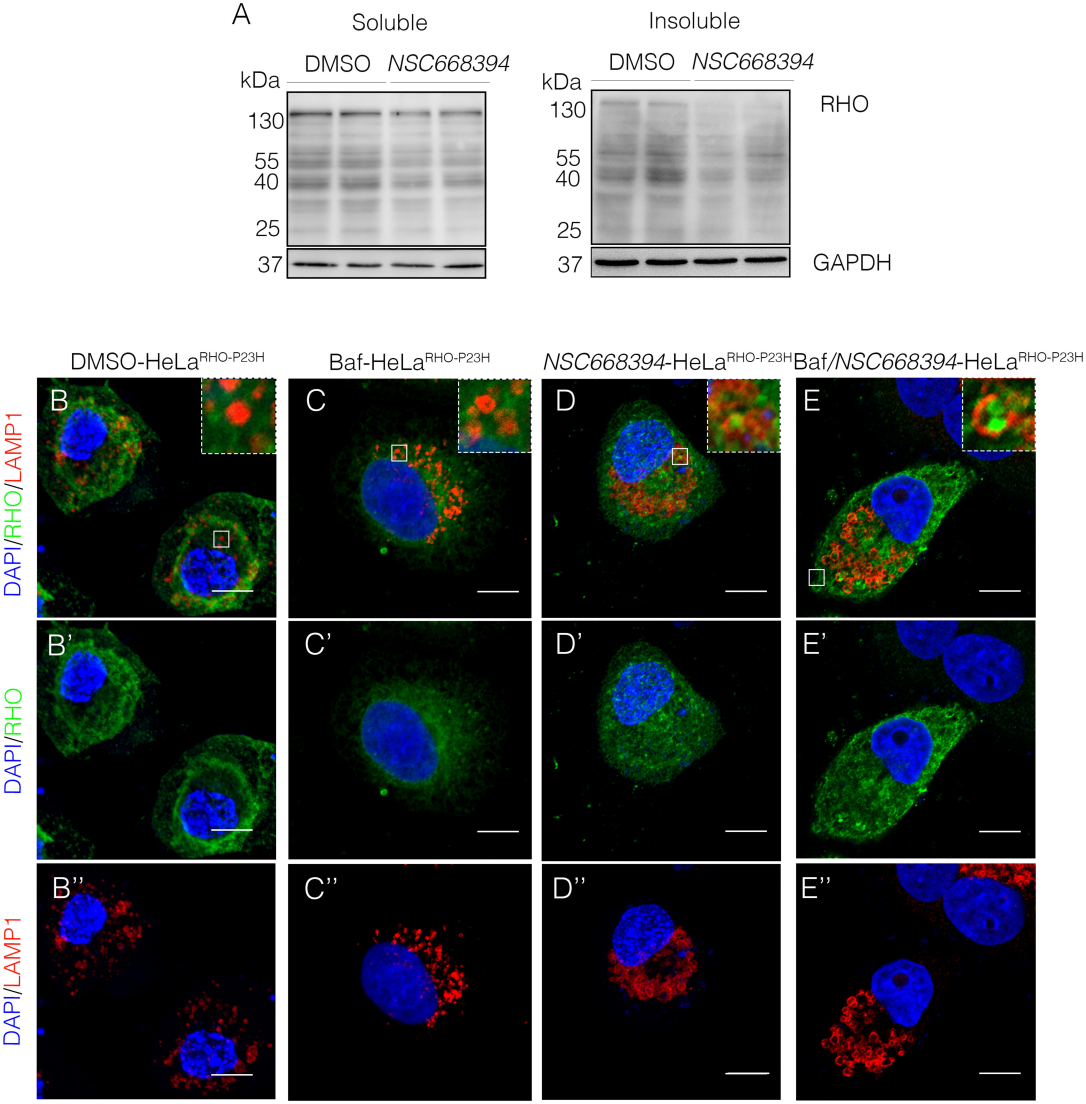
The *NSC668394*-treated HeLa^RHO−P23H^ cells show an autophagy-related reduction of protein accumulation. **(A)** Representative Western blot analysis of RHO protein in the soluble and insoluble fraction of HeLa^RHO−P23H^ cells treated with DMSO and *NSC668394*. Representative images of HeLa^RHO−P23H^ cells immunostained with anti-RHO and anti-LAMP1 from DMSO-treated **(B−B”)**, Baf-treated **(C−C”)**, *NSC668394*-treated **(D−D”)**, and Baf/*NSC668394*-treated **(E−E”)** HeLa^RHO−P23H^ cells. Nuclei are counterstained with DAPI (blue). Scale bar 10 μm.

**Figure 5:**
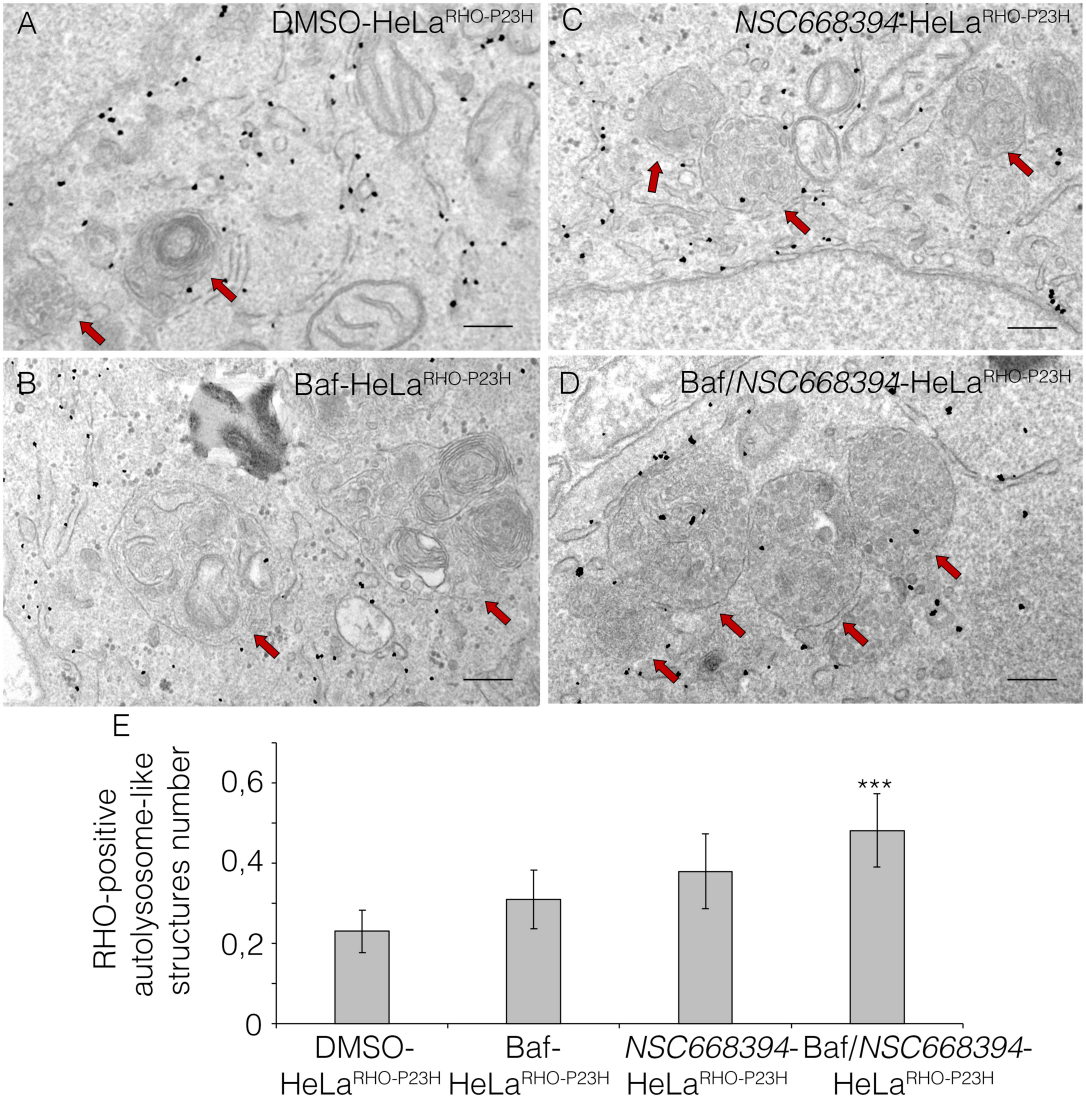
Ezrin inhibition induces RHO disposal *via* lysosomal activity. Immuno-electron microscopy (IEM) analysis of HeLa^RHO−P23H^ cells treated with DMSO **(A)**, Baf **(B)**, *NSC668394* **(C)**, and Baf/*NSC668394* **(D)**. Scale bar 250 nm. The RHO-positive autolysosome-like structures are indicated with red arrows. **(E)** The blot shows the quantification of the number of RHO-positive autolysosome-like structures. Bar graphs represent mean values ± SEM of independent experiments. ***p ≤ 0.005 *t*-test (Baf/*NSC66834*-HeLa^RHO−P23H^ *vs*. DMSO-HeLa^RHO−P23H^).

The recent discoveries have proven that lysosomes may reduce misfolded protein accumulation *via* activation of autophagy pathway (Mendes and Cheetham, 2008; Sakami et al., 2011). We sought to assess whether the protective effect of *NSC668394* is mediated by activation of lysosomal clearance of RHO^P23H^, we analyzed *NSC668394* administration on Hela cells overexpressing RHO^P23H^ (HeLa^RHO−P23H^). Interestingly, culturing HeLa^RHO−P23H^ cells in the presence of *NSC668394* led to a significant decrease of RHO^P23H^ in both insoluble and soluble fraction, as demonstrated by Western blot analyses, suggesting that there was a major change in rhodopsin degradation (Figure 4A). To further support that *NSC668394* treatment induced lysosomal clearance of RHO^P23H^, we assessed lysosomal cargo degradation on Ezrin-inhibited Hela RHO^P23H^ cells followed by a treatment with Bafilomycin A1 (Baf), a known inhibitor of vacuolar-type H(+)-ATPase that promotes alkalinization of the lysosomal lumen and inhibition of lysosomal cargo degradation, leading to an accumulation of undigested material within the lumen of already fused autolysosomes, and prevents new autophagosomes and lysosomes fusion (Yoshimori et al., 1991). As expected, treatment with Baf on Hela^RHO−P23H^ cells showed a slight increase of RHO^P23H^ accumulation in the lumen of autolysosomes (LAMP1-positive organelles) compared with DMSO-treated cells (Figures 4B–C”). Treatment with *NSC668394* on Hela^RHO−P23H^ cells increased the number of autolysosomes structures and showed a moderate increase of RHO^P23H^ accumulation in the lumen of autolysosomes (LAMP1-positive organelles) compared with DMSO-treated cells (Figures 4B–B”,D–D”). Notably, administration of Baf after 3 h of *NSC668394* treatment (Baf/*NSC668394*-treated Hela^RHO−P23H^) induced a high increase of autolysosomes structures containing RHO^P23H^ in the lumen, in accordance with a block of cargo degradation in already increased number of autolysosomes (Figures 4E–E”). These data were also confirmed in the 661W photoreceptor cell line (Supplementary Figure S3) (Wheway et al., 2019), supporting the notion that *NSC668394* treatment increases RHO^P23H^ degradation *via* autophagy pathway. Furthermore, to better investigate on rhodopsin sub-cellular localization, we performed an IEM analysis (Figure 5) and carried out morphometric issues. Hela RHO^P23H^ cells showed an increase of autolysosome-like structures, compared to normal levels, presumably due to the presence of the misfolded protein. Interestingly, the number and the volume of the autolysosome-like structures were even more increased in Baf/*NSC668394-*treated Hela^RHO−P23H^ cells (Figures 5A–D), confirming the treatment effectiveness. Most importantly, we found a significant increase in the number of rhodopsin-positive autolysosome-like structures in Baf/*NSC668394-*treated Hela^RHO−P23H^ cells (Figures 5A–E). Subsequently, we tested whether the reduction of RHO^P23H^ protein was also due to a block of protein synthesis or increase of proteasome activity. Remarkably, administration of *NSC668394* on Hela^RHO−P23H^ in the presence of cycloheximide (CHX) treatment, a well-known inhibitor of protein synthesis, further showed a faster degradation of RHO^P23H^ protein in *NSC668394-*treated cells compared with DMSO-treated control cells (Supplementary Figure S4A). Taken together, these data indicate that reduction of RHO^P23H^ protein in *NSC668394-*treated Hela^RHO−P23H^ is not due to an altered protein synthesis. To avoid the possibility that *NSC668394* might induce proteasome activity, we provide insights into how *NSC668394* regulates RHO^P23H^ clearance. To this end, *NSC668394*-treated HeLa^RHO−P23H^ cells were cultured in presence of the Bortezomib (BTZ), a proteasome inhibitor. Notably, following *NSC668394* treatment, a significant decrease of RHO^P23H^ was also detected upon BTZ conditions, as demonstrated by Western blot analysis (Supplementary Figure S4B). On the contrary, the levels of RHO^P23H^ protein were markedly increased in DMSO-treated HeLa^RHO−P23H^ cells upon BTZ conditions (Supplementary Figure S4B). Taken together, these data indicate that pharmacological *NSC668394* treatment specifically results in activation of lysosomal RHO^P23H^ degradation.

### *In vivo* Rescue of Retinal Degeneration Through Autophagy and ER-Stress Reduction

**Figure 6:**
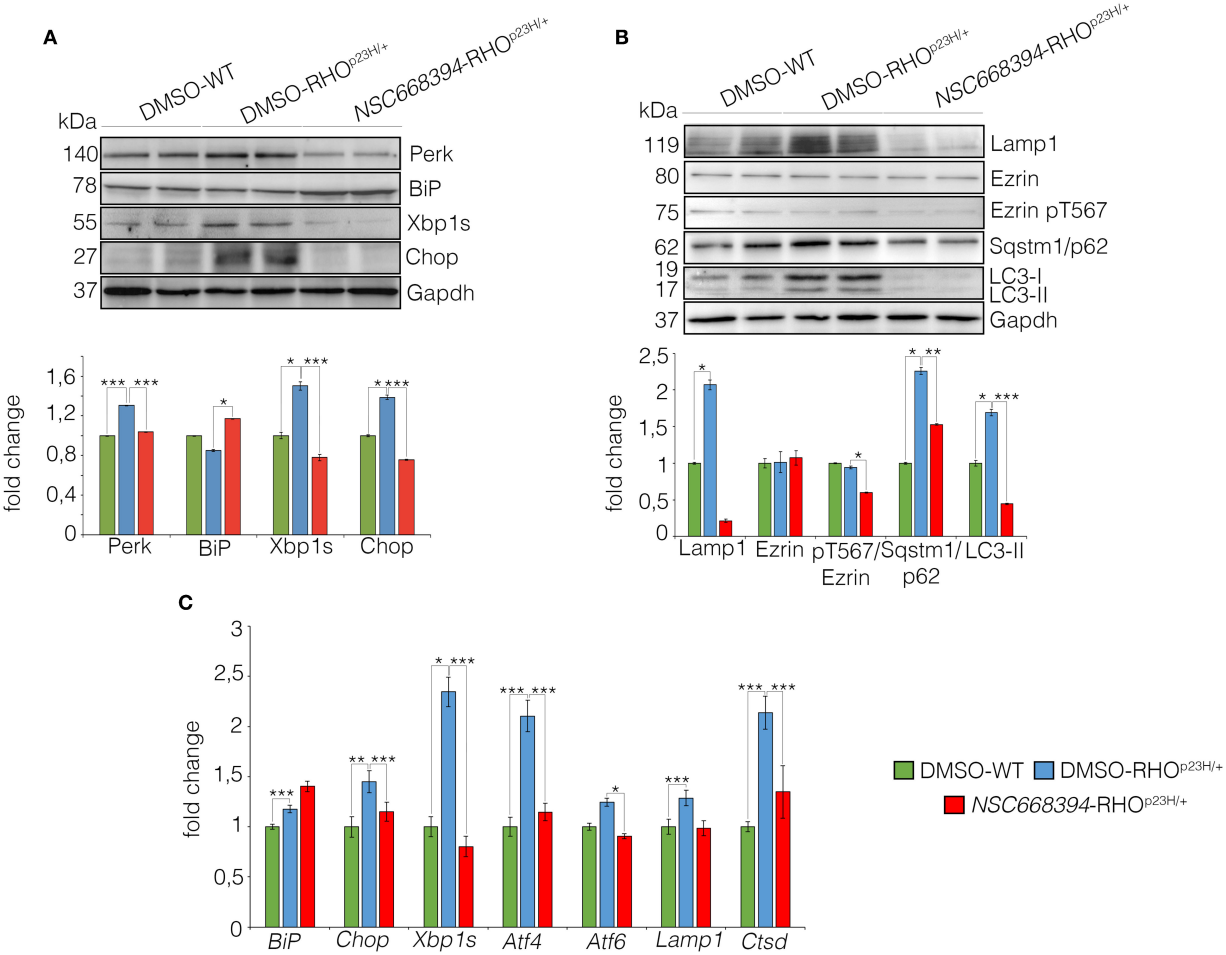
The *NSC668394* reduces ER-stress and autophagy in RHO^P23H/+^ mice. **(A)** Representative Western blot analysis of Perk, BiP, Xbp1s and Chop proteins from DMSO-treated WT, DMSO-treated and *NSC668394*-treated RHO^P23H/+^ mice at PN18. The plot shows the quantification of these proteins normalized to the Gapdh loading control. Bar graphs represent mean values ± SEM of independent experiments (at least *n* = 3 mice). **(B)** Representative Western blot analysis of Lamp1, Ezrin, Ezrin pT567, Sqstm1/p62, and LC3 proteins from retina of DMSO treated WT, DMSO-treated RHO^P23H/+^ and *NSC668394*-treated RHO^P23H/+^ mice at PN18. The plot shows the quantification of these proteins normalized to the Gapdh loading control. Bar graphs represent mean values ± SEM of independent experiments (at least *n* = 3 mice). **(C)** qRT-PCR assay for RPE of DMSO-treated WT, DMSO-treated RHO^P23H/+^, and *NSC668394*-treated RHO^P23H/+^ mice at PN18. The plot shows the expression level of *Bip, Chop, Xbp1s, Atf4, Atf6, Lamp1* and Ctsd normalized to the *Hprt* control. Bar graphs represent means values ± SEM of independent experiments (*n* = 3 mice). ***p ≤ 0.005, **p ≤ 0.01, *p ≤ 0.05 *t*-test (DMSO-RHO^P23H/+^ *vs*. DMSO-WT; *NSC668394*-RHO^P23H/+^ *vs*. DMSO-RHO^P23H/+^).

The mutation P23H rhodopsin was reported to activate ubiquitin/proteasome system (Chapple et al., 2001) and trigger the ER-stress response, which, in turn, lead to cell death (Griciuc et al., 2010). We therefore hypothesized that if *NSC668394* directly induces the clearance of RHO^P23H^ aggregates in the photoreceptor cells, the clearance of RHO^P23H^ misfolded protein should reduce ER-stress response *in vivo*. Notably, *NSC668394* treatment reduced the levels of Perk, Xbp1, and Chop proteins, commonly used as UPR markers, compared with vehicle-treated RHO^P23H/+^ mice at PN18 (Figure 6A). Consistent with this observation, we detected a significant increase in Bip protein level in *NSC668394-*RHO^P23H/+^ retina, as analyzed by Western blot analysis (Figure 6A). In addition, we observed reduced ER-stress and UPR markers, as demonstrated by *BiP, Chop*, and *Xbp1* gene expression levels in *NSC668394*-RHO^P23H/+^ compared with DMSO-WT and DMSO-RHO^P23H/+^ mice. Notably, *BiP* expression level was increased following *NSC668394* treatment, suggesting a possible BiP-mediated protective effect. Accordingly, *Chop* and *Xbp1* gene expression levels resulted significatively reduced in *NSC668394*-RHO^P23H/+^ mice compared with DMSO-RHO^P23H/+^ mice at PN18. Consistently, the expression levels of both *Atf4* and *Atf6* genes in *NSC668394*-RHO^P23H/+^ mice resulted similar to DMSO-WT mice, whereas DMSO-RHO^P23H/+^ mice showed higher *Atf4* and *Atf6* expression levels compared to DMSO-WT mice (Figure 6C). Taken together, these results strongly support that pharmacological *NSC663894* administration reduces both UPR and ER-stress pathways. Interestingly, RHO^P23H/+^ mice showed impairment of autophagy pathway as a consequence of RHO^P23H^-mediated ER-stress at the onset and during the progression of retinal degeneration in mice (Sizova et al., 2014; Qiu et al., 2019; Kakavand et al., 2020). We therefore hypothesized that if *NSC668394* treatment reduces ER-stress should re-establish and normalize autophagy pathway.

As expected, daily injection of *NSC668394* over 2 consecutive weeks reduced the active pT567-Ezrin protein level, without affecting Ezrin total protein level, and was efficient in rescuing imbalance of autophagy pathway in the retina of RHO^P23H/+^ mice when compared with vehicle-injected (DMSO) control animals (Figure 6B). Western blot analysis showed a normalization of Lamp1, SQSTM1/p62, and LC3-II protein levels, consistent with a rescue of autophagy pathway (Figure 6B). Interestingly, we also observed a reduction of LC3I. Accordingly, this was due to an increase of autophagic flux, leading to a rapid conversion of LC3-I in LC3-II, which is rapidly degraded.

Accordingly, retinas from *NSC668394-*RHO^P23H/+^ treated mice exhibited *Lamp1* and *Ctsd* expression levels similar to those detected in the retinas from DMSO-WT mice, supporting a normalization of autophagy pathway. Opposite results were observed in the retinas from RHO^P23H/+^ mice treated with DMSO, which exhibited increase of both *Lamp1* and *Ctsd* expression levels compared to DMSO-WT mice (Figure 6C), in line with previous results (Sizova et al., 2014). The ability of *NSC668394*to regulate differently autophagic flux was consistent with autophagic flux on RHO^P23H/+^ mice, compared with WT mice, that showed a rapid increase at PN6-PN12, followed by a reduction PN18-PN30 of autophagy, as showed by Western blot analysis (Supplementary Figure S4C).

Altogether, these data show that *NSC668394*-mediated activation of cell clearance exerts a protective effect in IRD model and may represent an innovative strategy to treat retinal dystrophies due to accumulation of misfolded protein.

## Discussion

Recently, autophagy inducers have gained increasing attention owing to its role in facilitating the degradation of insoluble protein aggregates protecting against their toxicity in neurodegenerative disorders in the central nervous system. However, there is little direct information on the forces driving autophagy pathway in retinal degeneration and how autophagy responds to misfolded protein accumulation in IRDs representing key questions that have been poorly addressed. Notably, translation of biological knowledge into pre-clinical and clinical studies for IRD remains challenging because there is a lack of autophagy inducers compounds specifically targeting autophagy pathway in the retina, which is finely modulated in PRs and RPE by light intensity. Recently, we demonstrated that daily induction of lysosomal biogenesis and function in RPE/PRs is post-transcriptionally regulated by a gene network employing the light-responsive miR-211, and its target gene, namely, Ezrin (Naso et al., 2020). Importantly, we demonstrated that pharmacological activation of lysosomal biogenesis and function, through *NSC668394*-mediated inhibition of Ezrin, rescued both lipofuscin accumulation and PRs degeneration in miR-211^−/−^ mice, pointing to a new lysosomal-based therapeutic intervention to treat retinal degeneration (Naso et al., 2020). Notably, Ezrin has also been validated as a key element influencing the critical steps of rhodopsin biogenesis, suggesting its role in controlling rhodopsin-laden membrane delivery to the rod outer segments (Deretic et al., 2004), but no role for Ezrin in clearance of rhodopsin has been reported thus far. The P23H mutation in rhodopsin is one of the most common causes of adRP, in which misfolded RHO^P23H^ is retained in the ER (Gorbatyuk et al., 2010). This event generates ER-stress that, in turn, activates autophagy pathway causing a secondary proteasome insufficiency and activation of cell death pathways. Therefore, a precocious remotion of RHO^P23H^ accumulation would alleviate ER-stress and consequently would prevent photoreceptors' cell death. Here, we hypothesized that early pharmacological inhibition of Ezrin would promote a pulsatile increase of autophagy for reducing misfolded RHO^P23H^ accumulation at the onset of its retention in the ER. This pharmacological treatment would prevent ER-stress and photoreceptors' cell death and rescue vision. Therefore, we investigated the systemic administration of *NSC668394* compound in RHO^P23H/+^ mice, in which misfolded rhodopsin protein, harboring the proline23-to-histidine mutation, accumulates in the ER.

Several studies have reported that the level of autophagy proteins resulted to be decreased in RHO^P23H/+^ rat retina, before the pathological signs of retinal degeneration, suggesting that an initial decline of autophagy pathway may contribute to the onset of the disease. On the other hand, the recent findings demonstrated that prolonged activation of autophagy participates in the deficiency of proteasome activity and activation of apoptosis at the onset of RHO^P23H/+^ pathological signs (Qiu et al., 2019). Therefore, the regulatory mechanisms controlling autophagy and its crosstalk among survival/cell death pathways need to be further investigated. We demonstrated that daily *NSC668394* treatment before the onset of the pathological accumulation of RHO^P23H^ may induce autophagy as a protective mechanism for eliminating and reducing misfolded RHO^P23H^ in the photoreceptor cells. Our data showed that administration of *NSC668394* compound rescues retinal phenotype (Figure 2, Supplementary Figure S1) and establishes normal ER-stress (Figure 6) preserving photoreceptors homeostasis and function, ultimately recovering visual function in RHO^P23H/+^ mice (Figure 1). The increased ERG responses (Figure 1) are mainly due to a clearance of RHO^P23H^ accumulation. This mechanism is corroborated by the reduction of both UPR and ER-stress pathways (Figure 6), accompanied by the decrease of TUNEL staining (Figure 3, Supplementary Figure S2) and a visible increase in ONL thickness in the retinas of *NSC668394*-treated RHO^P23H/+^ mice, at the analyzed stages (Figure 2, Supplementary Figure S1). In support of this mechanism, RHO^P23H^ aggregates were degraded within lysosomes upon *NSC668394* stimulation, *in vitro*, as detected by IEM analysis (Figure 5). The recent studies on therapeutic molecules for neurodegeneration has put forward several new candidates, including autophagy inducers in the neuronal cells (Silva et al., 2020). Thus, the observation that an increase in an autophagy-mediated clearance of aggregates in retinal cells could be not surprising, but there is still a lack of evidence for its efficacy. To our knowledge, our work is one of the first demonstration of pharmacological upregulation of autophagy may rescues phenotypes in IRD.

These findings support the notion that precocious intervention on RHO^P23H^ accumulation from PN6 onward, when pathological signs of disease are not evident in RHO^P23H/+^ retina, is highly effective and capable of halting the progression of retinal degeneration, preserving visual function. Thus, with the implication of the use of autophagy inducers to rescue multiple disease mechanisms common to genetically different pathologies, this study highlights that the time span within which potential treatments may positively modify the onset and progression of a disease need a more accurate attention.

In a preclinical trial study, the pharmacological therapy would ideally be applied in earlier disease stage, when pathological signs of altered molecular networks are not yet evident and detected. This may provide the best protective effect. This is particularly relevant for blindness condition, in which the detrimental molecular cascade leads to irreversible cell death of photoreceptor cells, and no useful vision remains to be rescued. Consistently, AAV-miR-211 delivery, a post-transcriptional repressor of Ezrin, was recently shown to preserve retinal function when injected before the onset of first pathological signs. Therapeutic rescue was associated with a reduction of apoptosis and amelioration of photoreceptor morphology in RHO^P347S^ mouse, suggesting that induction of autophagy can represent a mutation-independent therapeutic strategy to block disease onset and progression (Karali et al., 2020). In support of this notion, enhanced lysosomal activity by *NSC668394* also plays crucial role in lipofuscin degradation representing an attractive therapeutic target to treat Age-related macular degeneration and other related diseases (Naso et al., 2020).

Therefore, understanding the time span within which potential treatments may positive modifier and how the therapeutic use of autophagy inducers may be applied in the retina is still fundamental to move the field forward and provide clinical studies for evaluating the therapeutic efficacy of autophagy inducers in treating retinal diseases, in which toxic misfolded proteins accumulate.

## Data Availability Statement

The original contributions presented in the study are included in the article/Supplementary Material, further inquiries can be directed to the corresponding author/s.

## Ethics Statement

All studies on animals were conducted in strict accordance with the institutional guidelines for animal research and approved by Italian Ministry of Health; Department of Public Health, Animal Health, Nutrition and Food Safety in accordance with the law on animal experimentation (article 31; D.L. 26/2014; protocol number: 0016304-21/07/2020-DGSAF-MDS-P).

## Author Contributions

DI, GG, FN, SDG, FGS, EN, and EP performed the experiments and analyzed the data. DI and GG contributed to the experimental design, implementation, and interpretation. EN and FGS contributed to the technical part of the work. DI, GG, and IC conceived the experiments, analyzed the data, and wrote the manuscript. All authors read and approved the final manuscript.

## Funding

Work in the Conte group was supported by grants from the Million Dollar Bike Ride Grant Program MDBR-21-103-CHM, International Retinal Research Foundation, Italian Telethon Foundation TMICCBX16TT and MIUR FISR2020IP_03551, and Sanfilippo Children's Foundations and National MPS Society.

## Conflict of Interest

The authors declare that the research was conducted in the absence of any commercial or financial relationships that could be construed as a potential conflict of interest.

## Publisher's Note

All claims expressed in this article are solely those of the authors and do not necessarily represent those of their affiliated organizations, or those of the publisher, the editors and the reviewers. Any product that may be evaluated in this article, or claim that may be made by its manufacturer, is not guaranteed or endorsed by the publisher.

## Supplementary Material

Supplementary Figure S1 The *NSC668394*-treated RHO^P23H/+^ mice at PN18 show rescue of retinal phenotype. Representative images of retina cryosection immunostained with anti-Rhodopsin **(A,C)** and anti-*c*-Arrestin **(D,F)** antibodies from DMSO-treated WT **(A,D)**, DMSO-treated RHO^P23H/+^ **(B,E)** and *NSC668394*-treated RHO^P23H/+^ **(C,F)** mice at PN18. Nuclei are counterstained with DAPI (blue). At least *n* = 3 mice per group. Scale bar 10 μm. OS, outer segment; ONL, outer nuclear layer; INL, inner nuclear layer. **(G)** Graphs show the measure of ONL thickness from the retina of DMSO-treated WT (green line), DMSO-treated RHO^P23H/+^ (blue line) and *NSC668394*-treated RHO^P23H/+^ (red line) mice at PN18. **(H)** Graphs show the reduction of OS length from PN18 onward from each analyzed retina region of DMSO-treated WT (green line), DMSO-treated RHO^P23H/+^ (blue line) and *NSC668394*-treated RHO^P23H/+^ (red line) mice. **(I)** Graphs show normal cone morphology (cones/area) from the retina of DMSO-treated WT (green line), DMSO-treated RHO^P23H/+^ (blue line) and *NSC668394*-treated RHO^P23H/+^ (red line) mice at PN18. Error bars represent SEM. ^***^*p* ≤ 0.005, ^*^*p* ≤ 0.05 *t*-test (DMSO-RHO^P23H/+^ *vs*. DMSO-WT; *NSC668394*-RHO^P23H/+^ *vs*. DMSO-RHO^P23H/+^).

Supplementary Figure S2 The *NSC668394* treatment reduces TUNEL positive cells in PN18 RHO^P23H/+^ mice retina. Representative images of DMSO-treated WT **(A)**, DMSO-treated RHO^P23H/+^ **(B)** and *NSC668394*-treated RHO^P23H/+^ **(C)** retinas of PN18 mice stained with TUNEL-fluorescein. Positive cells are indicated with white arrows. Nuclei are counterstained with DAPI (blue). At least *n* = 3 mice per group. Scale bar 20 μm. ONL, outer nuclear layer; INL, inner nuclear layer; GCL, ganglion cell layer. **(D)** Graph shows the number of TUNEL positive cells from the dorsal and ventral retina of DMSO-treated WT, DMSO-treated RHO^P23H/+^ and NSC668394-treated RHO^P23H/+^ mice at PN18. Error bars represent SEM. ^***^*p* ≤ 0.005, ^**^*p* ≤ 0.01 *t*-test (DMSO-RHO^P23H/+^ *vs*. DMSO-WT; NSC668394-RHO^P23H/+^ *vs*. DMSO-RHO^P23H/+^).

Supplementary Figure S3 *NSC668394*-treated 661W^RHO−P23H^ cells show lysosomal clearance of RHO^P23H^. Representative images of 661W^RHO−P23H^ cells immunostained with anti-RHO and anti-LAMP1 from DMSO-treated **(A,A”)**; Baf-treated **(B,B”)**; *NSC668394*-treated **(C,C”)**; and Baf/*NSC66834*-treated **(D,D”)** HeLa^RHO−P23H^ cells. Nuclei are counterstained with DAPI (blue). Scale bar 10 μm.

Supplementary Figure S4 **(A)** Representative Western blot analysis of RHO protein from 0, 2, 4, and 6 h of CHX in DMSO and *NSC668394* conditions. The graph shows the quantification of RHO protein normalized to the GAPDH loading control. Bar graphs represent mean values ± SEM of independent experiments. **(B)** Representative Western blot analysis of RHO protein from 0 and 24 h of BTZ in DMSO and *NSC668394* conditions. The plot shows the quantification of RHO protein normalized to the GAPDH loading control. Bar graphs represent mean values ± SEM of independent experiments. ^***^*p* ≤ 0.005, ^**^*p* ≤ 0.01, ^*^*p* ≤ 0.05 *t*-test. (*NSC668394*-HeLa^RHO−P23H^ *vs*. DMSO-HeLa^RHO−P23H^). **(C)** Representative Western blot analysis of Lamp1, Sqstm1/p62 and LC3 proteins from retina of WT and RHO^P23H/+^ mice at PN6, PN12, PN18, and PN30. The plot shows the quantification of these proteins normalized to the Gapdh loading control. Bar Graphs represent means values ± SEM of independent experiments (*n* = 3 mice). ^***^*p* ≤ 0.005, ^**^*p* ≤ 0.01, ^*^*p* ≤ 0.05 *t*-test (RHO^P23H/+^ *vs*. WT).
