# Supplementary figures and images for: Induction of Autophagy Promotes Clearance of RHOP23H Aggregates and Protects From Retinal Degeneration
Source: Front Aging Neurosci. 2022 Jun 30;14:878958. doi: 10.3389/fnagi.2022.878958 (PMC9281868; doi:10.3389/fnagi.2022.878958)

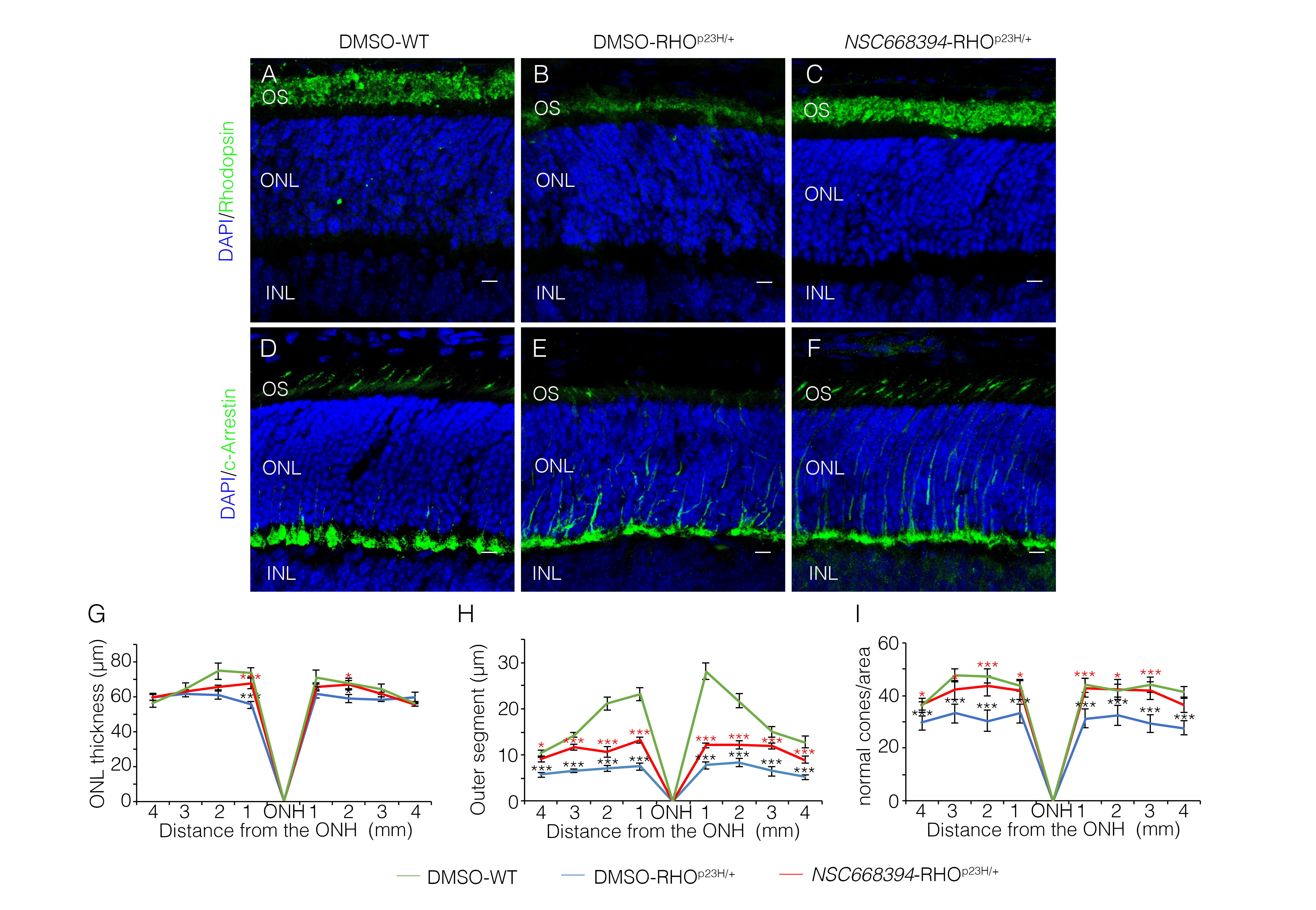

Supplement: Supplementary Figure S1 — The NSC668394-treated RHOP23H/+ mice at PN18 show rescue of retinal phenotype. Representative images of retina cryosection immunostained with anti-Rhodopsin (A,C) and anti-c-Arrestin (D,F) antibodies from DMSO-treated WT (A,D), DMSO-treated RHOP23H/+ (B,E) and NSC668394-treated RHOP23H/+ (C,F) mice at PN18. Nuclei are counterstained with DAPI (blue). At least n = 3 mice per group. Scale bar 10 μm. OS, outer segment; ONL, outer nuclear layer; INL, inner nuclear layer. (G) Graphs show the measure of ONL thickness from the retina of DMSO-treated WT (green line), DMSO-treated RHOP23H/+ (blue line) and NSC668394-treated RHOP23H/+ (red line) mice at PN18. (H) Graphs show the reduction of OS length from PN18 onward from each analyzed retina region of DMSO-treated WT (green line), DMSO-treated RHOP23H/+ (blue line) and NSC668394-treated RHOP23H/+ (red line) mice. (I) Graphs show normal cone morphology (cones/area) from the retina of DMSO-treated WT (green line), DMSO-treated RHOP23H/+ (blue line) and NSC668394-treated RHOP23H/+ (red line) mice at PN18. Error bars represent SEM. ***p ≤ 0.005, *p ≤ 0.05 t-test (DMSO-RHOP23H/+ vs. DMSO-WT; NSC668394-RHOP23H/+ vs. DMSO-RHOP23H/+). [file Image_1.TIF]

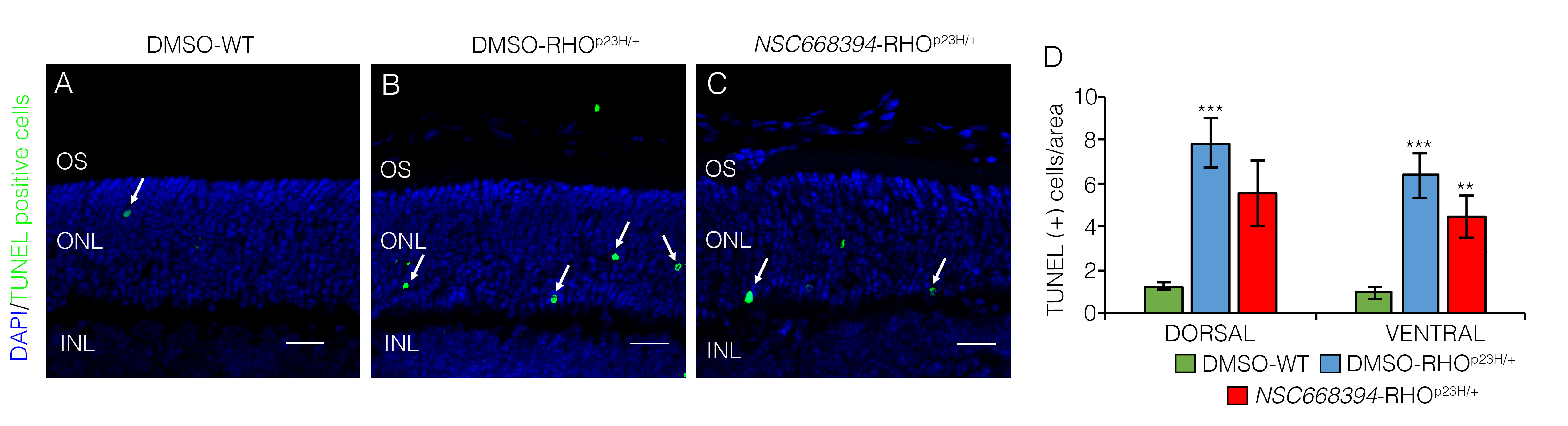

Supplement: Supplementary Figure S2 — The NSC668394 treatment reduces TUNEL positive cells in PN18 RHOP23H/+ mice retina. Representative images of DMSO-treated WT (A), DMSO-treated RHOP23H/+ (B) and NSC668394-treated RHOP23H/+ (C) retinas of PN18 mice stained with TUNEL-fluorescein. Positive cells are indicated with white arrows. Nuclei are counterstained with DAPI (blue). At least n = 3 mice per group. Scale bar 20 μm. ONL, outer nuclear layer; INL, inner nuclear layer; GCL, ganglion cell layer. (D) Graph shows the number of TUNEL positive cells from the dorsal and ventral retina of DMSO-treated WT, DMSO-treated RHOP23H/+ and NSC668394-treated RHOP23H/+ mice at PN18. Error bars represent SEM. ***p ≤ 0.005, **p ≤ 0.01 t-test (DMSO-RHOP23H/+ vs. DMSO-WT; NSC668394-RHOP23H/+ vs. DMSO-RHOP23H/+). [file Image_2.TIF]

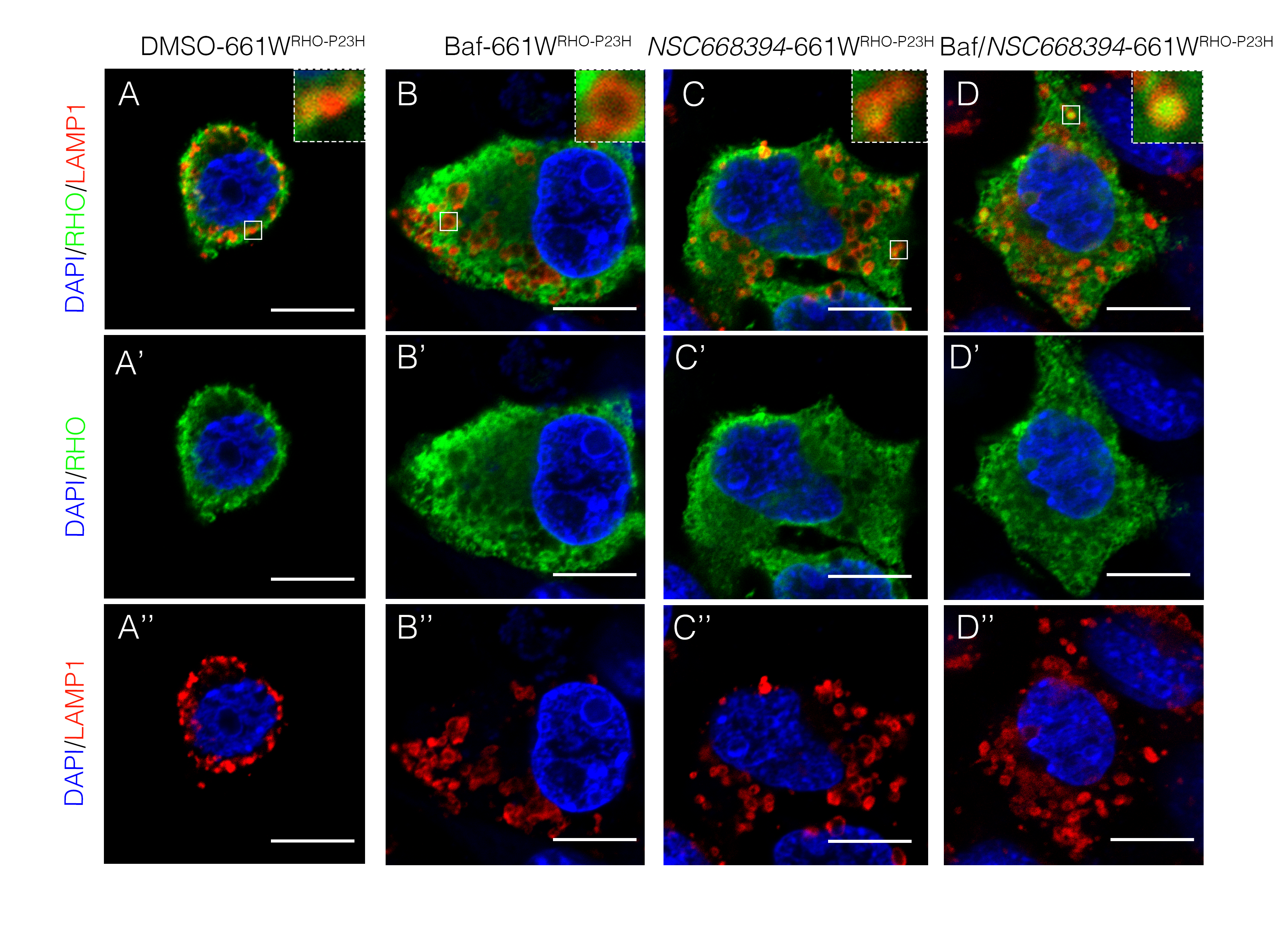

Supplement: Supplementary Figure S3 — NSC668394-treated 661WRHO−P23H cells show lysosomal clearance of RHOP23H. Representative images of 661WRHO−P23H cells immunostained with anti-RHO and anti-LAMP1 from DMSO-treated (A,A”); Baf-treated (B,B”); NSC668394-treated (C,C”); and Baf/NSC66834-treated (D,D”) HeLaRHO−P23H cells. Nuclei are counterstained with DAPI (blue). Scale bar 10 μm. [file Image_3.TIF]

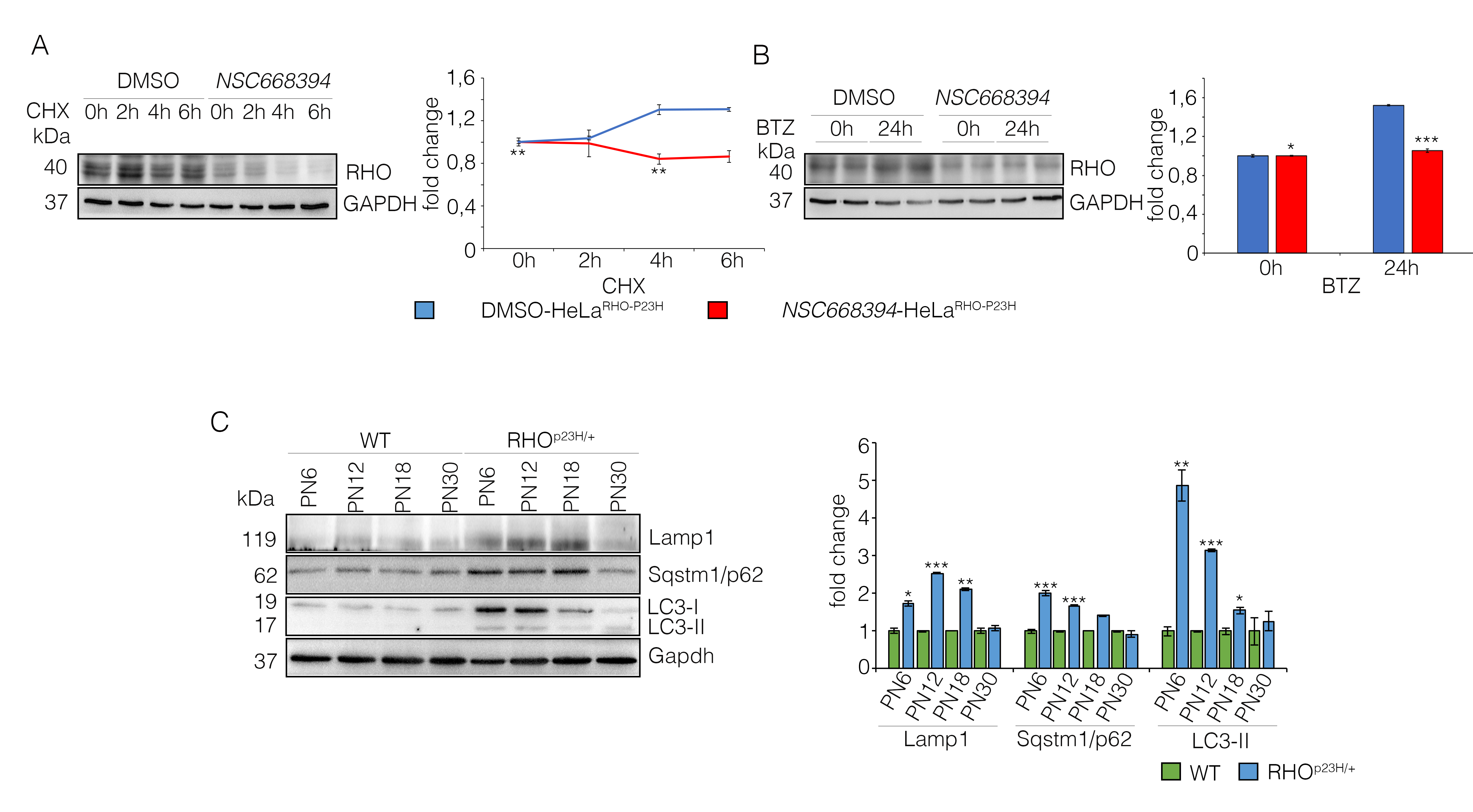

Supplement: Supplementary Figure S4 — (A) Representative Western blot analysis of RHO protein from 0, 2, 4, and 6 h of CHX in DMSO and NSC668394 conditions. The graph shows the quantification of RHO protein normalized to the GAPDH loading control. Bar graphs represent mean values ± SEM of independent experiments. (B) Representative Western blot analysis of RHO protein from 0 and 24 h of BTZ in DMSO and NSC668394 conditions. The plot shows the quantification of RHO protein normalized to the GAPDH loading control. Bar graphs represent mean values ± SEM of independent experiments. ***p ≤ 0.005, **p ≤ 0.01, *p ≤ 0.05 t-test. (NSC668394-HeLaRHO−P23H vs. DMSO-HeLaRHO−P23H). (C) Representative Western blot analysis of Lamp1, Sqstm1/p62 and LC3 proteins from retina of WT and RHOP23H/+ mice at PN6, PN12, PN18, and PN30. The plot shows the quantification of these proteins normalized to the Gapdh loading control. Bar Graphs represent means values ± SEM of independent experiments (n = 3 mice). ***p ≤ 0.005, **p ≤ 0.01, *p ≤ 0.05 t-test (RHOP23H/+ vs. WT). [file Image_4.tif]
